# Editorial: Perinatal Mental Health: Expanding the Focus to the Family Context

**DOI:** 10.3389/fpsyt.2021.719053

**Published:** 2021-07-19

**Authors:** Susan Garthus-Niegel, Yael Benyamini, Antje Horsch

**Affiliations:** ^1^Department of Medicine, Faculty of Medicine, Medical School Hamburg, Hamburg, Germany; ^2^Faculty of Medicine Carl Gustav Carus, Institute and Policlinic of Occupational and Social Medicine, Technische Universität Dresden, Dresden, Germany; ^3^Department of Child Health and Development, Norwegian Institute of Public Health, Oslo, Norway; ^4^Bob Shapell School of Social Work, Tel Aviv University, Tel Aviv, Israel; ^5^Institute of Higher Education and Research in Healthcare-IUFRS, Faculty of Biology and Medicine, University of Lausanne, Lausanne, Switzerland; ^6^Department Woman-Mother-Child, Faculty of Biology and Medicine, Lausanne University Hospital, Lausanne, Switzerland

**Keywords:** perinatal period, childbirth, mental health, family, partner, parents, couple relationship, parent-child relationship

In the past two decades, the interest in psychosocial aspects of pregnancy and childbirth, such as perinatal mental health has sharply increased. Much of the research on perinatal mental health has focused on depression and anxiety, fear of childbirth, and post-traumatic stress during pregnancy and following childbirth. The vast majority of this body of research focused only on mothers, despite the fact that these experiences usually take place within a family.

This Research Topic therefore set out to expand the focus by adding the perspectives of fathers/partners, as well as the infant, thus englobing the whole family. Here, we present a collection of articles, which together will help researchers and clinicians, on the one hand, to learn how the perinatal context affects parental mental health and, on the other hand, how perinatal mental health issues may affect the couple relationship, as well as the infant and the parent-infant relationship. Furthermore, relevant issues related to the assessment of perinatal mental health problems are discussed. We believe that this article collection may help to better understand the mental health needs of (future) parents during the perinatal period, and may help to identify risk and protective factors for perinatal mental health problems. In turn, this knowledge may facilitate the development of ways of supporting families from pregnancy to postpartum, including evidence-based interventions aimed at prevention and/or treatment.

This Research Topic includes 29 articles. Twenty-four of them are based on original research, coming from many different countries. Almost all of these countries are located in Europe, North America, or Australia; one study is from South Africa. A few of the studies focused on minority populations within their countries, such as Aboriginal parents in Australia (Chamberlain et al.) or fathers from traditional mid to low socio-economic backgrounds in South Africa (Crowley et al.). There are also five review articles: Three were based mainly on studies from Europe and North America and only a few from Asia and Africa; only one review (Mojahed et al.) on the prevalence of intimate partner violence during the perinatal period included a more geographically and culturally diverse set of studies.

Among the original research articles, most were quantitative, only two were qualitative, and one combined quantitative and qualitative data. It is notable that most of the quantitative studies (n = 14) were longitudinal. Twenty-one of these articles described empirical studies, mostly based on data collected from parents: Seven studies investigated fathers, one investigated mothers, and three investigated parents of both genders, but not in couples. In contrast, fewer studies were designed to examine dyadic processes between parents (six studies) and/or between parents and their children (three studies). Regarding all 29 articles in our Research Topic, two studies focused only on the prenatal period, 16 focused only on the postpartum period, and 11 spanned both time periods.

The articles in this Research Topic pertain to three main themes: (1) parental mental health and parenting during the perinatal period; (2) the couple relationship in the perinatal context; and (3) the implications for the infant, parenting, and the parent-infant relationship. We will briefly review the contributions regarding each theme, followed by several contributions related to assessment in this research field, and conclude with some future directions (see Figure 1).

**Figure 1:**
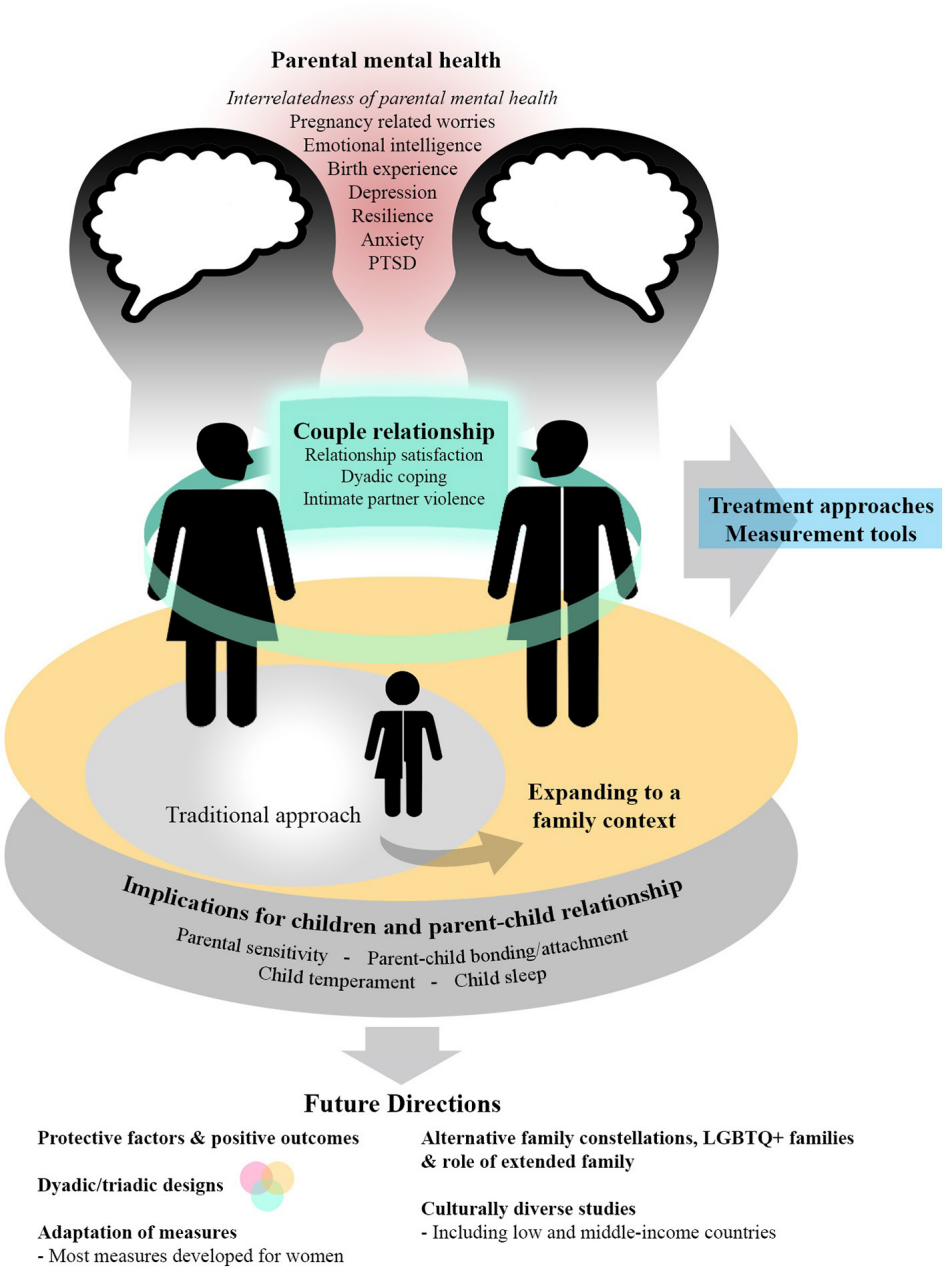
An integrative depiction of themes covered in this Research Topic including future directions.

## Parental Mental Health and Parenting During the Perinatal Period

### Depression

A systematic review and meta-analysis by Thiel et al. concluded that paternal and maternal depression were positively correlated across the perinatal period, concurrently and prospectively, in studies ranging from a few weeks to 1 year postpartum. However, regarding the longer-term impact, Walker et al. reported that fathers and children aged 11–12 years seemed to be affected only to a small extent by maternal postpartum depression or anxiety symptoms. Garthus-Niegel et al. found that rates of paternal depression symptoms decreased from 9% during pregnancy to 5% at 8 weeks postpartum and identified four latent depression profiles for expectant fathers. Perceived social support and relationship satisfaction appeared to be protective against paternal depression symptoms. According to Bamishigbin et al., greater amount of time spent with the infant, parenting self-efficacy, and material support were all significantly associated with lower levels of paternal depressive symptoms during the first year. Focusing on parents of very low or extremely low birthweight babies, Neri et al. found higher levels of postpartum depression among parents with extremely low birth weight babies at 3 months postpartum compared to parents of babies in other birth weight groups. However, later in the first year postpartum, those rates of postnatal depression stabilized in all groups, with mothers' and fathers' scores correlating at each time point. Investigating predictors of maternal depression, deMontigny et al. reported positive cross-sectional correlations between perceived paternal involvement and both dyadic adjustment and parental alliance and negative correlations between parenting alliance and parenting stress, as well as mothers' depression.

### Pregnancy-Related Worries

Göbel et al. found that overall worries were rather low in expectant fathers. However, they proposed that identifying those reporting major worries beyond health- and birth-related aspects might support their psychosocial adjustment.

### Childbirth-Related Post-Traumatic Stress

In a prospective cohort study, Schobinger et al. found higher maternal than paternal prevalence rates of childbirth-related acute stress disorder (CB-ASD) as well as childbirth-related post-traumatic stress disorder (CB-PTSD). Focusing on parents of very low birthweight (VLBW) infants aged 5 years, Barthel et al. found that none of the parents fulfilled the criteria for a diagnosis of CB-PTSD. Postnatal CB-PTSD symptoms and a VLBW preterm birth predicted maternal CB-PTSD symptoms at 5 years postpartum, whereas psychiatric lifetime diagnosis and postnatal CB-PTSS predicted paternal CB-PTSD symptoms.

As mentioned above, historically, both researchers' and professionals' attention has focused on *maternal* depressive or posttraumatic symptoms during the perinatal period. The current collection of articles shows that fathers may also be prone to these reactions, particularly in the face of well-recognized stressors such as a VLBW infant or lack of social support. Moreover, the findings reported in this collection show how mothers' and fathers' responses are related and how each parent's mental health, stress, parental alliance, and involvement may affect their partners' mental health. The findings also suggest protective factors, as well as factors that can help to identify mothers, fathers, and couples at risk for maladjustment during the transition to parenthood.

## The Couple Relationship in the Perinatal Context

The findings of Alves et al. indicated complex prospective interactions between maternal and paternal psychosocial adjustment and their dyadic coping over time, suggesting that dyadic coping strategies should be integrated into the support that expectant couples receive in order to strengthen the “parental team”. Complex patterns of changes in emotional intelligence around childbirth in couples were shown in another dyadic analysis, by Galdiolo et al. When one partner's emotional intelligence scores decreased, the other parent's scores increased. This again reinforces the idea of childbirth requiring a “team approach” by the parents, so that one parent can compensate for the other's difficulty in the emotional management of parenting. Knappe et al. reported that although the overall relationship quality, as reported by fathers, remained relatively stable during the perinatal period, fathers with comorbid anxiety and depressive disorders reported lower partnership satisfaction at postpartum. Furthermore, antenatal father-to-child attachment, as well as self-reported ante- and postnatal partnership quality in fathers, were positively related to postnatal father-to-child attachment. Finally, focusing on the extreme end of partner relationship difficulties, Mojahed et al. in their narrative review reported that psychological violence was the most prevalent form of violence during the entire perinatal period. Interestingly, the few studies on *bidirectional* intimate partner violence mostly found that women's perpetration was almost as high as that of their partner or even higher. However, these findings need to be interpreted with caution, and not only the occurrence, but also the motivations and the contexts of the bidirectionality of intimate partner violence need to be considered. Mojahed et al. concluded that intimate partner violence is highly prevalent during the entire perinatal period, particularly in populations suffering from social inequalities. Taken together, these articles underscore the importance of viewing and supporting parents as a dyad: The transition to parenthood often places much strain on the relationship, risking its stability and quality; a dyadic approach can optimally utilize dyadic coping strategies and enable compensatory efforts within the couple, when one is experiencing greater difficulty than the other.

## Implications for the Child, Parenting, and the Parent-Child Relationship

Stuijfzand et al. found that maternal PTSD-CB symptoms at 1 month postpartum were negatively prospectively associated with mother-infant bonding at 3 months postpartum, although this effect disappeared after adjusting for maternal psychological distress at 1 month postpartum. No such effects were found for fathers, thus highlighting gender-specific paths. In another prospective community sample, Holopainen et al. reported that women's birth experience was related to both mothers' and their partners' parenting stress, but not to child attachment, neither directly nor indirectly. Macdonald et al. found that fathers who reported more symptoms of depression and anger were more likely to report weaker bonding with their infant and more co-parenting problems, as well as less perceived social support. In a lab study by Kazmierczak et al. using a crying life-like doll, men's, but not women's, history of maltreatment in childhood was related to them perceiving their partner as less empathic, which led to lower parental sensitivity of such couples. Little and Sockol reported that new parents who had experienced parental divorce or separation in their family of origin did not differ from those from intact families with regard to romantic relationship satisfaction, parent-infant bonding, attachment anxiety, or attachment avoidance. Attachment anxiety and avoidance were both associated with romantic relationship dissatisfaction and greater impairment in the parent-infant bond. Investigating the developmental change of parents' perception of their daughters' and sons' temperament and its association with parental mental health problems, Sechi et al. found that mothers and fathers gave similar descriptions of their child's temperament throughout the first year of life; however, infant temperament showed developmental changes, as well as gender differences. Foley et al. showed that maternal and paternal talk about their infant differed in their associations with parental well-being, couple relationship quality, and caregiving sensitivity. Therefore, new mothers and fathers may benefit from distinct strategies to foster attention to their developing infant. One such approach for fathers was explored by Cowley et al., here fathers participated in a Baby Theater, with trained actors modeling sensitive and responsive interactions. Results showed that this could be a promising intervention to encourage fathers' involvement with their babies, particularly in a patriarchal society.

A systematic review by Knappe et al. showed that parental cognitions, particularly those about difficulties with limit-setting, often preceded child sleep problems. Parental cognitions thus seem to be an important factor in the development and maintenance of infant sleep problems. In their case study, Singh et al. described an infant mental health day-clinic treatment based on forming a triangle of co-regulation between clinician, parent, and infant to first help the parent and then the infant with persistent crying and sleep problems settle down.

Overall, these articles show that both parents' mental health and well-being may be relevant to parenting behavior and thus to parents' relationships with their infant. However, several studies found differing associations for mothers and fathers, suggesting gender-specific pathways. These differential results once more stress the importance of considering both parents' perspective. Intervention research and clinical practice aimed at promoting warm and empathetic parent-infant relationships should therefore take a gender-sensitive approach.

## Assessment Issues/Measurement Tools of Perinatal Mental Health

Employing a concept analysis and a Delphi survey, Van Haeken et al. examined the concept of perinatal resilience during the first 1,000 days of life. Perinatal resilience was described as a circular process toward greater well-being in the form of personal growth, family balance, adaptation, or acceptance, when faced with stressors, challenges, or adversity during the perinatal period. Social support, sense of mastery, self-efficacy, and self-esteem were thought to enhance the capacity to be resilient and to potentially prevent perinatal mental health problems. Chamberlain et al. collected the views of predominantly Aboriginal stakeholders regarding the relative importance of domains proposed for complex trauma assessment and opinions on how to conduct these sensitive discussions with Aboriginal parents. Although most of the participants thought it was important to assess the proposed complex trauma domains with Aboriginal parents, the authors concluded that assessments to identify Aboriginal parents experiencing complex trauma should only be considered when the conditions of safety, trusting relationships, respect, compassion, adequate care, and capacity to respond were assured.

In their mixed methods evidence synthesis, Darwin et al. summarized the evidence on the performance of mental health screening tools and the acceptability of mental health assessment, specifically in relation to fathers, other co-parents, and partners in the perinatal period. They highlighted a paucity of research on the assessment of the mental health of fathers, co-mothers, step-parents, and other partners in the perinatal period. Siew et al. reviewed the application and performance of tools assessing the father-infant relationship from pregnancy to 24-months postpartum, in the context of parental psychopathology and infant outcomes. They found 38 unique tools, most of which were originally developed for mothers. The authors concluded that much remains to be learned in terms of the validity of such instruments and their adaptation and suitability to different ages, outcomes, and cultural contexts. Vermeulen et al. described the development, as well as face and content validation of the Belgian DDads (Depression in Dads) questionnaire aimed at identifying the awareness, knowledge, and attitudes of the general population toward paternal perinatal depression. This new questionnaire may help with informing stakeholders, such as policy makers and healthcare professionals, to identify gaps and predisposed attitudes in society toward paternal depression, both of which may be barriers for appropriate care. Finally, in their opinion piece, Baldoni and Giannotti called for the integration of appropriate, not mother-centered, screening of at-risk fathers as an essential prerequisite for perinatal health services, given the impact of psychological distress on maternal health, family adaptation, and child development.

## Conclusion and Future Directions

This Research Topic of 29 articles set out to expand the traditional approach in perinatal mental health research — which focusses predominantly on mothers — to the family context. The studies in this collection examined the role of the perinatal context for parental mental health, as well as the implications perinatal mental health may have for the couple, the child, and the parent-child relationship.

Most of the included studies focused on risk factors, mental health problems, or constructs with negative emotional valence in general. For the future, it would be highly interesting to also conduct studies on constructs with positive emotional valence, e.g., on protective factors and positive outcomes.

Further, only a few studies examined dyadic processes. As a future direction, we would like to see an increased use of dyadic designs in perinatal mental health research. Clearly, there is interdependence between partners who are raising a child together. In the framework of the Actor-Partner Interdependence Model [APIM; (1, 2)], for instance, such interdependence can be accounted for. With the inclusion of child data, even triadic designs would be conceivable in the perinatal research field.

Another methodological aspect relates to the use of appropriate measures in perinatal mental health research. Originally, most of the commonly used measures were developed for women. In terms of a gender-sensitive approach and with regard to other populations, such as fathers, these measures need to be revalidated or even adapted to the respective target group.

Also, while this article collection expanded the traditional approach in perinatal mental health research to some degree, further expansion to also include alternative family constellations or LGBTQ families would be timely. For instance, instead of traditionally conceptualizing parents as ≪ mother ≫ and ≪ father ≫, some families would prefer to refer to them as ≪ birthing parent ≫ and ≪ co-parent ≫. In addition, it would be interesting to expand the focus from the nuclear family and to investigate the role of the extended family, such as grandparents, as well.

Finally, as is the case in most other perinatal mental health research, the studies in this Research Topic mainly stemmed from Europe, North America, or Australia. Therefore, regarding future directions, we would like to call for more culturally diverse studies, including studies from low and middle-income countries. One example for such research is the recently launched INTERSECT project aimed at investigating “cross-cultural information on the prevalence of postpartum PTSD, as well as cross-cultural variation in the etiology and manifestation of childbirth-related PTSD worldwide” (https://blogs.city.ac.uk/intersect/). The cultural context is particularly important in this area, as different cultures may differ in their expectations regarding motherhood and fatherhood, which may have implications for their adjustment along the transition to parenthood and for their relationship along this transition.

## Author Contributions

SG-N initiated the Research Topic. SG-N, YB, and AH were topic editors and wrote the manuscript. All authors contributed to manuscript revision, read, and approved the submitted version.

## Conflict of Interest

The authors declare that the research was conducted in the absence of any commercial or financial relationships that could be construed as a potential conflict of interest.
